# Successful adaptation of a MinION nanopore for protein sequencing

**DOI:** 10.1093/synbio/ysae017

**Published:** 2024-11-08

**Authors:** Casey-Tyler Berezin

**Affiliations:** Department of Chemical and Biological Engineering, Colorado State University, 1370 Campus Delivery, Fort Collins, CO 80526, USA

DNA sequences may store the genetic code, but the diversity in protein sequences, composed of over 20 amino acids and numerous post-translational modifications (PTMs), far surpasses that of DNA with its four nucleotides. The complexity of the proteome underscores the variation in biological functions and disease states, making the ability to sequence and characterize proteins in their native form a long sought after aspiration. While DNA sequencing has continued to improve for over 50 years [1–4], incremental advancements in nanopore-based protein sequencing have facilitated only the successful sequencing of short peptides and DNA–protein conjugates [5–7]. Now, a team of researchers led by Prof. Jeff Nivala at the University of Washington in the USA has developed an approach for sequencing intact folded, modified proteins using the MinION flow cell, originally developed by Oxford Nanopore Technologies for DNA sequencing [8, 9]. The work will accelerate therapeutics development and the use of protein barcoding for screening protein libraries, as well as significantly improve our ability to validate synthetic proteins for materials development, drug delivery, genetic sensors, and more.

A series of proteins for amino acid sequencing through optimized regions (PASTORs) were designed to develop the technology. Each PASTOR contains a negatively charged N-terminus that allows the protein to be threaded through the nanopore into the positively charged *trans* compartment of the flow cell by electrophoretic force. The key to accurate sequencing, however, lies at the C-terminus. Beyond a stably folded blocking domain and positively charged repeat that prevent the protein from fully translocating is an ssrA tag that will be bound by a ClpX motor. Once the protein is threaded into the pore, the motor is loaded and its unfoldase and translocase activity slowly pulls the protein through the pore in an ATP-dependent manner.

As with DNA sequencing, each amino acid makes a distinct current trace as it moves through the nanopore. By carefully introducing mutations into PASTOR sequences, the researchers resolved each of the 20 standard amino acids and developed a model for discriminating between residues based on their current signatures. Smaller amino acids generally let more current through the pore than larger ones, while charged amino acids showed variable currents due to either pressure or resistance to re-enter the negative *cis* compartment. A random forest modeling approach achieved a 28% accuracy rate classifying amino acids among all 20 candidates, and the correct residue was in the top-8 guesses 81% of the time. To improve the accuracy of protein sequencing, a rereading mechanism was implemented by introducing a proline-rich ‘slippery’ sequence near the N-terminus. At this sequence, the motor momentarily loses its grip on the protein, allowing it to rethread by electrophoretic force and then rebind the motor at the ssrA tag, initiating the sequencing process again. This simple change increased the model’s accuracy to 61% in the 20-way classification task and led the top-7 accuracy to reach 99%.

Once the standard amino acids could be reliably discriminated, the researchers successfully characterized the currents associated with single and double phosphorylation events by two different kinases. In addition, they challenged the nanopore with proteins containing tertiary structure. Although these folded domains could be electrically unfolded and loaded, they naturally refolded in the *trans* compartment, leaving the motor to repeatedly try to pull the bulky protein through. By introducing easily detectable point mutations to destabilize the tertiary structure, unfolding and translocation occurred more reliably and folded PASTORs could be accurately sequenced.

Optimization is needed to expand this methodology for the characterization of native proteins. Perhaps most importantly, the basecalling model must be expanded for *de novo* amino acid determination. In addition, the need to append synthetic sequences to both termini is a barrier. However, this can be achieved through existing terminus-specific chemical-conjugation techniques, which could be combined with additional optimization steps to develop a reliable large-scale sample preparation protocol. In fact, addition of the ssrA tag post-protein extraction would reduce endogenous degradation as well as increase the conversion efficiency (the number of translocation events compared to the number of pores) above the optimized rate of 27%. Identifying optimal pore variants for protein sequencing should also increase conversion efficiencies. Nevertheless, the ability to accurately sequence folded amino acid sequences with PTMs using a straightforward protocol represents a great advancement in protein sequencing that will refine our ability to characterize native proteins and develop synthetic proteins to transform our understanding of life and disease.
